# Revisiting Keynes in the Light of the Transition to Circular Economy

**DOI:** 10.1007/s43615-021-00016-1

**Published:** 2021-04-08

**Authors:** Patrizia Ghisellini, Renato Passaro, Sergio Ulgiati

**Affiliations:** 1 Department of Sciences and Technologies, Parthenope University of Naples, 80143 Naples, Italy; 2 Department of Engineering, Parthenope University of Naples, 80143 Naples, Italy; 3 School of Environment, Beijing Normal University, Beijing, China

**Keywords:** Keynes, Circular economy, Expansionary fiscal policies, Green new deal

## Abstract

The study briefly recalls the evolution and crisis of the theory and thought of John Maynard Keynes with the rise and progressive dominance of the neoliberalism paradigm. The exercise has been made for evaluating the effects of such process for the sustainability of the global economy and society. In this view, we explored how Keynes’ contribution could be useful for the global economy in building a new paradigm of socio-economic development underpinning the transition to circular economy (CE). We also evaluated the adoption of the Global Green New Deal including a case study of Italy for the purpose of suggesting how that topical political programme can be key in the CE transition. Given the urgency of environmental problems, we underline the importance of the adoption of Keynesian expansionary “green mission oriented” fiscal policies with the purpose of allowing the triggering of a virtuous circle of sustainable welfare involving the Green New deal and the transition to CE. At the basis of such virtuous circle, we propose a new paradigm based on a revisited Keynesian paradigm and models of economy within the framework of Genovesi’s “civil economy” that entails an active role and responsibility of all the societal actors (consumers, companies and institutions). In this view, the “spirit” of Keynes in the economy, policy and society could be appreciated once more and be extremely useful along with other scholars’ contributions in accelerating the CE transition and a more sustainable development.

## Introduction

Unprecedented and severe environmental problems such as resource scarcity and climate change are challenging worldwide economies, societal assets and well-being [1]. The Royal Swedish Academy of Science pointed out that “we can no longer discuss our economic future without considering the enormous costs of climate change” [2].

This provides further confirmation to the fact that climate change is strongly affecting the global economy and points out an urgent need to reduce the environmental costs of production and consumption activities [3] and better integrate the environmental issues in economic theories and practices [4, 5].

Since the last century the environmental awareness towards such problems has progressively increased as showed by the recent mobilisation of the civil society, media and the attention of Governments and institutions [6] also in this Covid-19 emergence [7].

However, the political efforts should be much higher, in particular in developed countries, towards implementing strategies that tackle the anthropogenic climate change, as well as for building resilience to the most severe impacts of climate change and environmental degradation [8, 9].

In this perspective sustainable patterns “must be sought on many different levels, and given the magnitude of the challenges the effects must be significant compared to the existing situation” [10].

In the range of solutions and constraints, we would emphasise the importance of defining and framing the development model underpinning the circular economy (CE), coherently with other authors involved in this endeavour [11–15]. Jackson [16] points out that in developed countries the quantitative economic growth is no more needed to achieve well-being as Governments can achieve full employment, low inequality and reduction of greenhouse gas emissions by changing the current linear model of economic growth. At this regard, given the increasing constraints posed by the natural environment, the role of public intervention in developed countries should be oriented in the promotion of new values and concepts at the basis of the growth model that are less consuming for the natural resources [17]^1^ given that a continued quantitative economic growth cannot be taken for granted and be limited by e.g., resource scarcity [13]. Since the Rio Earth Summit (1992) it is clear that what is needed to move in this direction is an integrative and shared policy framework for sustainable development at national, subnational and international level capable of putting into practice strategies, programmes and initiatives based on public intervention and aimed at environmentally sound and sustainable economic growth and development in both developing and industrialised countries [18].

Indeed, the current and dominant linear model of development, at the global level, mainly oriented to the goal of a quantitative economic growth is based on the economic philosophy of neoliberalism [19] or new liberalism [20] succeeded progressively since 1980s to the Keynesian philosophy^2^. The latter influenced strongly public thinking and policies in particular after the Second World War and was based on Keynes’s thought focused on the analysis of some of the causes of market failures at macro level and on macroeconomic policies as remedies to such failures. In this view, it supported expansionary fiscal policies favouring aggregate demand and the propensity to consume [21].

Currently, for the latter issue, Keynes’s thought could be perceived and labelled as a representative of the linear model of production and consumption [10] and in general as an “influencer” of consumerism after an economic crisis [22]. To show this issue, we cite a recent article appeared on an Italian Newspaper last year after the lockdown. The author stated that: “I have no doubt. If John Maynard Keynes was alive, he would appear on TV to incite us to spend as much as possible as he did on the radio during the ‘Great Recession’”. The article recalled the Keynes thinking with the purpose of evidencing the increase of savings of Italian families during the lockdown and the importance of the Government to revitalise consumption and supporting the recovery of the real economy [22].

We underline that Keynes’ promotion of consumption is associated to a particular historical period. No doubt Keynes’ theory of consumption supported the economic growth model in quantitative terms. He did not explore to a sufficient extent the environmental consequences of policies promoting the propensity to consume in spite of some of his papers started highlighting the rise of environmental problems linked to industrial growth around the 20s of the last century. However, his contribution should not be restricted to an issue of consumption in view of searching causes to contemporary challenges and solutions for the transition to sustainable development [23]. Keynes contributed to evidence some of the limits of a market economy by finding solutions to the economic crisis and giving a new vigorous stimulus to the public intervention in social protection as an essential base for the modern welfare state in Europe [24]. As a result, the value of his contribution (including the theory of consumption) can be better understood only by means of an historical analysis involving the genesis of his theories (the Great Depression on 1929), the period of their wider application in political economy (after the Second World War) [25] as well as the progressive replacement from the 70s with the rise of new theoretical models including the “monetarism” by [26] and later the rational expectations by [27], the neo-institutional view [28] and in general of a new paradigm (neoliberalism) [24]. Nowadays, the latter is the dominant conceptual framework that governs the global economy [29].

In this study, as a starting point of our research work, we will briefly explore the evolution of Keynesian thinking and paradigm. This overview is a base for building an original work focused on evidencing how the contribution by Keynes could be useful in the development of a new paradigm underpinning the CE. In this perspective, we explore the so-called “Global Green New Deal” or “Green Deal” launched by the European Union^3^ as a kind of expansionary Keynesian fiscal policies. It identifies an orientation in public policies [8, 30–32] for the development of a green economy. In this study, we emphasise such topical expansionary fiscal policies to accelerate the current CE transition. In that framework, we also explore the case of Italy, where the past Government announced that the Green Deal implementation in the country will reorient the whole productive systems [33].

On the bases of these premises, the structure of the paper is the following: the next section deals with the conceptual framework of the paper and with materials and methods used to analyse the data and information that we considered for the literature review of Keynes thinking and the case study of Global and Italian Green Deal. Moreover, this section briefly outlines the genesis and main features of Keynes’s theory, the adoption of Keynesian thinking in the post-World War II period as well as its progressive decline with the rise of the neoliberalism paradigm. The “Revisiting the Keynesian Paradigm in the Light of the Current Circular Economy Transition” section, in view of the progressive need for replacing the neoliberalism paradigm, revisit some aspects of the Keynes’ theory along with the contribution of recent theories more focused on the responsibility of societal actors such as citizens and companies as a mean for overcoming the limits of public choices. The “Discussion” section deals with the potential contribution by Keynes for building and accelerating the transition to a new political, economic and social paradigm embedding the move to CE as well as discusses the main components of the Global and Italian Green Deal. Finally, the “Conclusions” section concludes the study by summarising the key findings.

## Conceptual Framework

As evidenced previously, the study takes as a basis the analysis of the fundamentals of Keynes’ theory that signed, in particular in the post-World II War, the development of Keynesian paradigm in the economy, policy and society of industrialised countries. We also compared such paradigm with the paradigm of neoliberalism that currently dominates in the global society. In that, the main elements of Keynesian theory have been summarised by relying on his seminal work “The General Theory of Money, Interest and Employment” [34] and existing several macroeconomics contributes whereas the evolution of the Keynesian paradigm has been investigated by performing an initial search on Web of Science^4^ with the following keywords:

- Keynes AND new liberalism (period 1950–2020): 7 results (2 articles selected);
- Keynes AND neoliberalism (time period 1950–2020): 16 results (2 articles selected);
- Keynes AND sustainable development (2000–2020): 16 results (2 articles selected);

The relative low number of searched articles could be due to the fact that the investigated relationships are more concerned to political interests compared to the scientific research as well as that the topics are of recent evidence as showed by the date of publication of most of the studies that are after the years 2010. This is the period following the economic crisis of 2008.

The search has been further expanded on the search engine “Google” where the same keywords have been used along with others such as “Keynesian paradigm and neoliberalism”, “neoliberalism”, “new liberalism”, “neoliberalism and welfare state”. Other searches have been performed on various Italian libraries, publishers and databanks as well as using cited articles from searched and selected articles. The main aim for the selection of the studies were the need for highlighting the main features of both paradigms and their differences also in the perspective of the existing multiple global challenges related to environmental and social problems as well as CE transition.

The study also briefly evaluates the features and development of the Green Deal at the global level whereas in more detail the Italian Green Deal. For the purpose, the most recent data related to Global Green New Deal and in particular the data of renewable sources development coming from the IRENA last reports (years 2020; 2019; 2017) have been collected. The Italian Green Deal has been analysed by relying on documents of the Italian Government and Ministry of Environment, Territory and Seas, Ministry of Economic Development and Italian Advanced Institute of Environmental Protection and Research (ISPRA).

### Genesis of Keynes’ Theory Within the Challenge of Great Depression

The most important Keynes’ work, “The general Theory of Employment, Interest and Money” [34] has been published during the Great Depression, that continue to be considered by the historians the most dramatic economic downturn [35], that shocked and affected the western capitalist countries (in particular US and Germany) in the late 1920s in a way that is unprecedented compared to other previous and subsequent crisis in the capitalist industrial system [36].

In such work, Keynes underlined the importance of the adoption in an economic crisis of anticyclical compensatory policies [36]. The crisis developed for about 10 years from 1929 to 1939 and was characterised by steep declines in the Gross Domestic Product (GDP) and industrial production, deflation*,* mass unemployment, banking panics, and sharp increases in rates of poverty and homelessness [25, 35]. In that period, the initial and weak measures of welfare state provided a few supports to the population [36]. The economic and social data about the magnitude of the crisis are particularly shocking and do not need comments to be understood: “In the United States, where the effects of the depression were generally worst, between 1929 and 1933 industrial production fell nearly 47 percent, GDP declined by 30 percent, and unemployment reached more than 20 percent. By comparison, during the Great Recession of 2007–09, the second largest economic downturn in U.S. history, GDP declined by 4.3 percent, and unemployment reached slightly less than 10 percent” [35].

It is not surprising that Keynes focused more on potential solutions to such impressive decline than to their environmental consequences. On the other hand, the environmental issue was “culturally” absent from the public opinion panorama and from the economic and political debate. Nevertheless, the analysis of Keynes thought to free trade reveals his awareness and critique towards the model of continued economic growth without limits as in one of his writings he wrote that: “The same rule of self-destructive financial calculation governs every walk of life. We destroy the beauty of the countryside because of the unappropriated splendours of nature have no economic value. We are capable of shutting off the sun and the stars because they do no pay a dividend …. Or again, we have until recently conceived it a moral duty to ruin the tillers of the soil and destroy the age-long human traditions attendant on husbandry if we could get a loaf of bread thereby a tenth of a penny cheaper” [23].

The research work by Keynes was mainly centred in building a theory that explained the determination of the GDP and the level of unemployment. The absence of full employment of production factors and the arbitrary and unfair distribution of income and wealth were considered by Keynes the most relevant failures of the market economy [37].

Keynes’ contribution [34] deeply affected the mainstream classical thinking that based on the Say’s law (1803) considered that production generates its own a same amount of aggregate demand. The Say’s law, based on the “laissez-faire” thinking, focused on the capacity of a capitalist system to naturally approach full employment and prosperity without government intervention in the long run [38]. On the contrary, Keynes proposed the adoption of Governmental policies to support the aggregate demand as in the short run the demand affects the aggregate production (Blanchard, 2000).

He showed that the normal case in an economy is the equilibrium of “underemployment” and that the volume of employment (or unemployment) depends on the level of effective people demand of goods and services. This evidences that e.g. the level of employment is decided on the market of goods and not in the market of labour as assumed by the classical economists [37].^5^ The level of income depends on the amount of the aggregated demand that is the sum of consumption (private and public) and investments. In turn, consumption and the multiplier effect of the investments into the income depends on the propensity to consume (the more consumption grows, the more income increases). Indeed, according to Keynes, given that consumption generated by the private demand has a decreasing trend in industrialised countries due to the increasing savings propensity, the private consumption is insufficient in the short run to sustain the aggregate production [39].

Consequently, given that the level of income of full employment is not achievable by changes of the production factors prices (wages and interests), Keynes suggests that the increase of income and employment is possible only by an increase of the aggregate demand and in particular by an expansion of public spending and other policies aimed to redistribute wealth and support consumption and investments [37]. In that, public spending acts as an anticyclical factor in the economy [25].

### Development and Crisis of Keynesian Paradigm

After the Second World War, many countries in America, East Asia and Europe (including Italy) experienced and enjoyed a period of high economic growth and employments rates as well as of higher well-being [11]. The economic paradigm underpinning such good social and economic performances was centred on the advances of technological progress, wide adoption of labour division and the development of mass consumption markets thanks to the favourable prices of consumption goods. In this period the technological progress and exploitation of scales economies also lead to an increase of the size of the companies. In that, the high growth rate of aggregate supply was sustained by a relatively stable growth rate of aggregate demand. This last was achieved by both the consolidation of welfare state and the progressive State intervention in the economy as well as by the adoption of anticyclical Keynesian policies. The latter contributed to reduce the level of uncertainty in the economy encouraging the investments in plants and machineries encouraged by public investments. Moreover, income policies assured the positive adjustment of real wages following the achievements in the technological progress [21].

The progressive deregulation of the international trade increased the amount of exported manufactured goods from industrialised countries. The unemployment rate reduced in the post-war period due to the increase in the productivity of labour and in such way it would be possible to reach the full employment rate at the beginning of the 60s. This increased further wages, unit cost of labour as well as inflation. However, the adoption of policies aimed to reduce the rate of inflation were not able to achieve such goal and maintaining the full employment. These problems experienced by industrialised countries at different times caused at the beginning of the 70’ the collapse of the Bretton Woods Agreements and the entire system of governance of the international economy [40].

The philosophy underlying the Agreements was based on the experience of the Great Depression of 1930s which features showed the need for regulating both the local and international markets. The monetary system introduced by Bretton Woods stated the rules for commercial and financial relations among the participating countries (USA, Canada, Western European countries, Australia and Japan) based on the central role of the US currency. The monetary system created was a gold exchange standard, with fixed exchange rates between currencies, all pegged to the dollar, which in turn was pegged to gold. The progressive trade liberalisation has been one of the pillars of the Agreements which functioning should comply with other international agreements promoted by the United Nations and its divisions (such as UNESCO, ILO, OMS, UNEP, UNDP) with the purpose of achieving social, humanitarian, health and environmental goals [21].

The collapse of Bretton Woods Agreements remarkably changed the principles of the governance of the global development as well as within the national economies in the coming years. The economic and political paradigm progressively moved from the Keynesian model to a completely different paradigm (so-called neoliberalism) incorporating the theories and research of scholars of the Chicago School of Economics such as Friedman [26] and later Lucas [27]. The neoliberalism paradigm was centred on the market as the best institutional form of organisation of economic activities for the achievement of individual and social welfare [40, 41] and on monetarism that involved in policies a strict control of the inflation [21]. Palley [19] evidences that in industrialised countries, in neoliberalism period, the economic agenda has been dominated by policies following the “U.S. model” that involved: “deregulation of financial markets, privatization, weakening of institutions of social protection, weakening of labor unions and labor market protections, shrinking of government, cutting of top tax rates, opening of international goods and capital markets, and abandonment of full employment goals, all under the guise of the natural rate. International economic policy has been dominated by the ‘Washington Consensus,’ which advocates privatization, free trade, export-led growth, financial capital mobility, deregulated labor markets, and policies of macroeconomic austerity” [19]. These policies produced devastating effects in some cases (e.g., financial crisis in Mexico, South-East Asia, Brazil, Russia, Argentina) and an increase of the unemployment and poverty [21, 42].

A general criticism that is made to the Keynesian approach lies in the fact that the market demand is determined from above by an invisible hand that knows the needs of people better than people themselves. The Keynesian paradigm was criticised from within by other neo-classical theory-oriented models which contributed to evidence the limits of the market. The neo-institutional theory highlighted that the market failures caused by externalities (e.g. air and water pollution are negative externalities) [43], asymmetric information (in turn due to uncertainty, opportunism, and bounded rationality of the individuals) and monopolies [44] can be tackled by direct bargaining between private actors, vertical integration [45], collaborative relationships among firms and the formation of social capital based on interpersonal and social trust [46]. In this view, Westlund [47] highlighted the key role played by civil society and social capital, which resides in institution (industry, public body) and other organisations. Indeed, the neo-institutional view also represents a critic to the neoliberalism approach whereas in the former the environment (and the market) is regulated by a set of social and legal norms and rules instead that pure market mechanisms.

Another criticism about the market failure notion has been represented by the public choice school of economics. The latter has had great impact on contemporary reforms of the public sector, replacing the Keynesian economics logics themselves that expanded the public service sector. The public choice approach has led to seeking to replace governments with markets to challenge or remedy market failure [48].

In social practice, further critics have been raised by James [49] in his book where he questions the capacity of capitalism to have fulfilled its expectations made in the early 1980s. As a matter of fact, the achievement of a pleasant life filled with beautiful things paved the way with “anxiety, job insecurity, frustration, difficulty getting to the end of the month, human relations deteriorated and by the pressures of an increasingly devoted to consumerism”. These words well capture the perspective offered by the new liberalism having its emphasis on the absolute autonomy of the individual that rejects any idea of responsibility “of one individual toward the other, of the State toward individuals, and even of individuals towards themselves” [50].

## Revisiting the Keynesian Paradigm in the Light of the Current Circular Economy Transition

Within the history of economic thought, the Keynesian can be considered an evolution of the classical liberalism thought. Instead, the neoliberalism seems break both with the Keynesian and the classical liberalism [51]. At the beginning the neoliberalism claimed to revitalise from the classical one^6^ in response to the excessive deviation of the Keynesian liberalism from the classical approach [21]. However, later on, even not repudiating and sharing some features with the classical liberalism it has been unable to sustain the critical assessment of the classical tradition “without changing quite fundamentally their understanding of the relationship between society, economy, and state” [20].

A period of coevolution of both Keynesian and neoliberalism in political models can be observed before the 1980s. In that, the genesis and development of environmental policies at the global level are one of the effects of the coevolution with the Keynesian thought and theories. From the 1980s the neoliberalism progressively dominated at cultural, economic and political level leading to a deregulation of the markets, privatisation and dismantling of services provided within the welfare state [19, 21, 40] as well as an increase of the political power of the banks and the relative weight of finance in individual economies [51].

It is now established that the great international economic crisis of the dot-com bubble in the late 1990s and the recession of 2008 were caused by an excess of deregulation, but many more are the phenomena that today put at risk the global economic system dominated by the neoliberalism: the huge increase in income and wealth inequalities, the pollution that is causing the climate change, a reduced welfare system, the high increase in the size of businesses [40, 52–54].

**Fig. 1:**
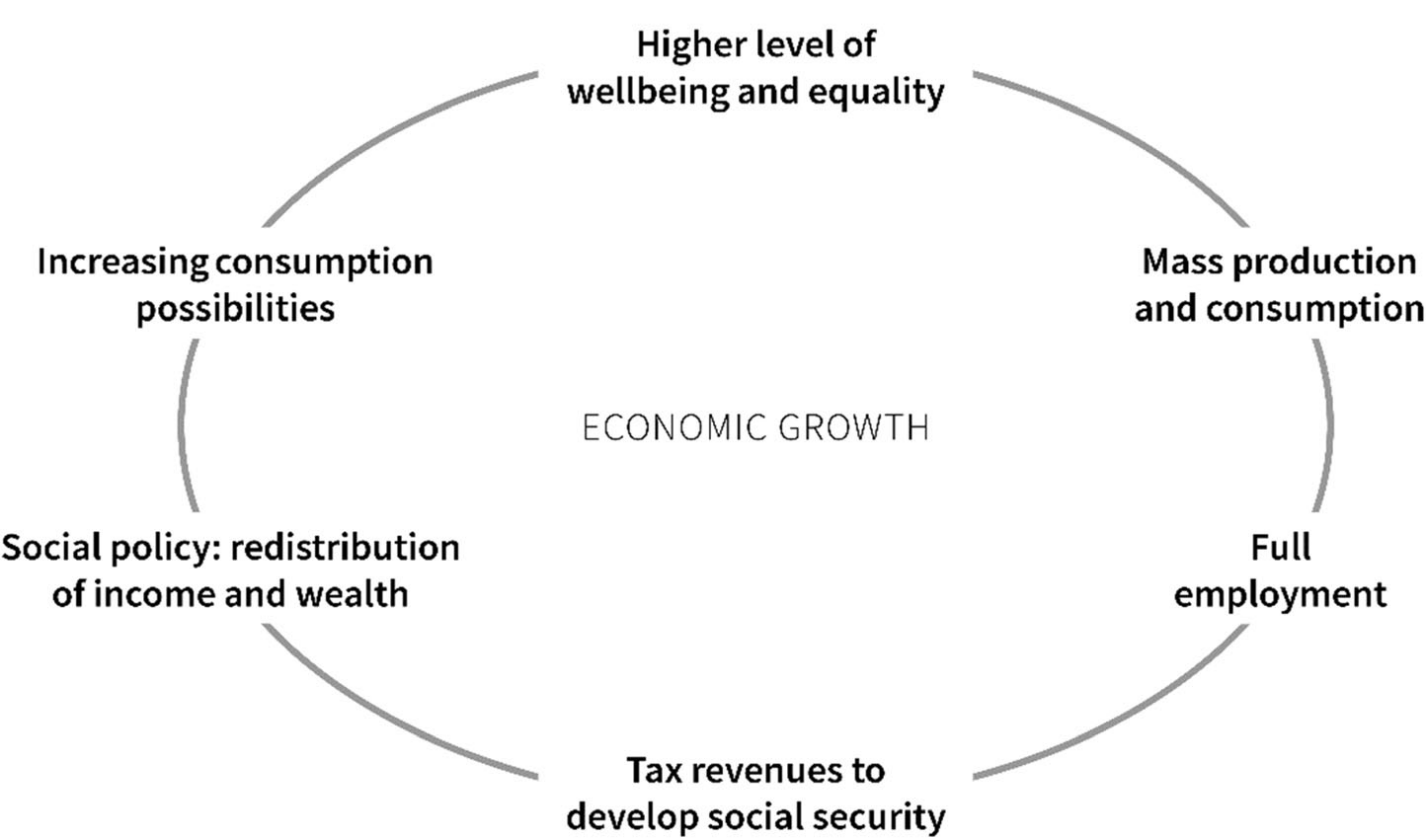
Traditional virtuous cycle of welfare state (Post-World War II) stimulated by Keynesian fiscal policies. Source: [11] (included in an open access article distributed under the Creative Commons Attribution License which permits unrestricted use, distribution, and reproduction in any medium, provided the original work is properly cited)

The presence of inefficiencies in the form of environmental externalities and uneven distribution of wealth in current economy and society should increase the awareness on the limits of the markets in satisfying only part of economic and social needs. This does not imply that the cures to the market failures should only find a solution in more public intervention [40, 55]. The abuses that occurred in this regard in the 1960s translated into cases of conflict of interest and excessive bureaucracy in the provision of public services. These are still current problems that affect negatively the global economies. Consequently, an excess of deregulation should not be followed by an excess of State intervention [40]. However, the role of the State should be active and not insignificant (being one of the factors of development) [56] as also evidenced by classical economists such as Smith [57] and provide the needed institutional framework for the economy to operate as well as deliver the important services for the well-being of the society [24, 40]. In that, as evidenced in the “Development and Crisis of Keynesian Paradigm” section, after the post II World War, the adoption of Keynesian fiscal policies in industrialised countries have been key factors of development and of an increased well-being allowing the switch off of the dynamo of continued GDP growth in the traditional virtuous circle of welfare state (Fig. 1) [11].

**Fig. 2:**
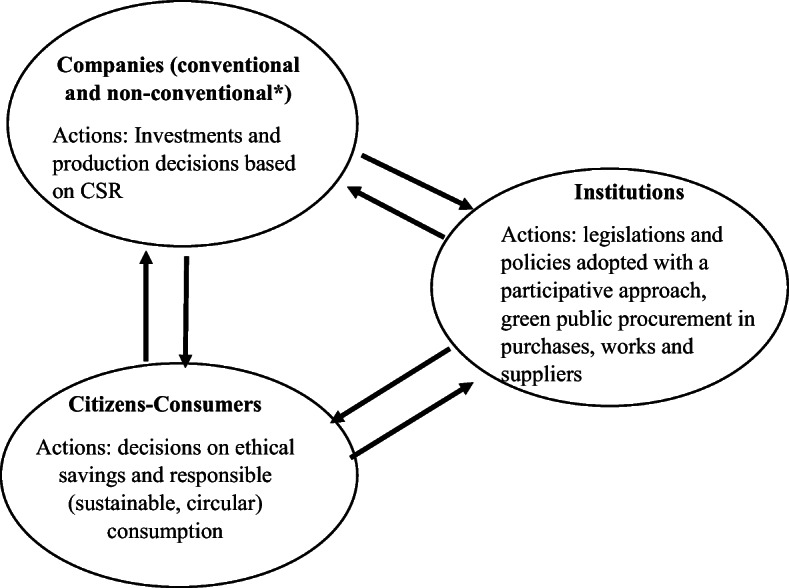
The virtuous cycle based on the principle of civil responsibility of consumers, companies and institutions. Adapted from [59]. Note: *non-conventional forms of companies include benefit corporations, cooperatives, non-profit companies, foundations, social companies

In this view, the current emphasis would be on revisiting the Keynesian liberalism in light of the historical political facts [21] and current multiple challenges along with the development of alternative models under the umbrella of the so-called “civil economy” [58] where the economic agents and in particular consumers [59] play an active role in the society^7^ in terms of positive responsibility of their environmental and social impacts [59]. The responsibility of all societal actors (consumers, companies and institutions) is the key driver in such model of economy (Fig. 2) both in the creation and maintenance of the continuous improvement of a virtuous cycle towards a better well-being in the economic system.

Consumer responsibility implies that consumers should take into account the environmental costs and impacts of their consumption patterns and lifestyles^8^ and be aware that consumption choices on products have an environmental and ethical impact [60]. Such choices have the power of reshaping the market and production practices in a more sustainable (and circular) manner. In this view, these actions agree with Flannery [61] who considers citizens as “the weathermakers”, and he argues fittingly that the “future (and we could also add ‘the present’) ...hangs on our actions”.

The market power of consumption is also recognised in the CE action plan of the European Commission [62] that underlines the importance of integrating the consumer’s perspective within the CE transition and the adoption of measures and assessment (e.g.: environmental labelling) tools to orient consumers’ choices towards sustainable products and services [62]. This contributes to green and reorient the transition to the CE in the European economy towards alternative growth models as well as improve the functioning of the markets by designing out negative externalities [63]. More broadly, at the global level “sustainable consumption and production patterns” are included in the Millennium Sustainable Development Goals UNEP 12.

The role of company’s responsibility reflects into the governance of environmental and social impacts in the achievement of economic goals. The adoption of Corporate Social Responsibility (CSR) strategies are aimed to the improvement of the environmental performances in terms of more cleaner and sustainable products and processes as well as that take into account the impacts on wellbeing of all the stakeholders (e.g. employees, local and global communities, animal welfare etc.). The attention to stakeholders’ impacts date backs to the seminal research work “Strategic Management: A stakeholder approach” by Freeman [64] who defined the stakeholders as “any group or individual who can affect or is affected by the achievement of the organisation’s objectives” [64, 65]. It is interesting to note that the management of the impacts to the stakeholders by the companies involves the allocation of resources to CSR strategies with the final goal of achieving “win-win” results, in the medium-long run [66]. On the other hand, it does not mean that managers can manipulate stakeholders to reach the most favourable trade-offs. “Stakeholder management aims to allocate resources adequately given the outcome of prospective scenarios; it also aims to repair previous damages traditionally left to society’s kind-heartedness, which are referred to as ‘externalities [67]”.

The strategies and goals of CSR can be introduced in the conventional companies mainly perceiving goals of economic efficiency (profits) or by modifying their legal framework to achieve other goals than economic efficiency as in the case of benefit corporations or economy of communion, born in Brazil on the experience by Chiara Lubic on 1991 [68]. The first commits to create public benefits and sustainable value in addition to profits (e.g. agree to have lower profits to favour the social climate and relationships with the employees) whereas the economy of communion requires the companies to split the profits into three parts: development of the company, cultural training of employees and support to poor people. Moreover, other legal forms of companies perceiving economic and social goals taking into account of their environmental impacts are the cooperatives, social companies, non-profit companies and foundations [40].

This orientation towards the institution of civil responsibility as a bottom-up approach provides the stimulus to governments towards the importance of the social responsibility [59, 69, 70] and forces them to consider or reconsider better the claims coming from the civil society in legislations, policies and practices e.g. Green Public Procurement. The latter is a voluntary tool for Europe’s public authorities by which they can actively contribute to the increase of sustainable production and consumption patterns and circular economy. Their market power is relevant (if measured in terms of purchases of services, works and supplies by public authorities accounting for 14% of GDP in the EU every year).

In general, the implementation of such a virtuous system creates a regenerative circle based on a higher responsibility of the subjects and a new order of values (e.g., informal institutions) as key factors for a new model of a more sustainable development 71. The institution of civil responsibility has been also supported as a tool in EU environmental policy with the V Environmental Programme adopted after the Rio Conference (1992) on Sustainable Development. In the V Programme the emphasis is on the concept of “shared responsibility” that requires a more active involvement of all economic actors including public authorities, public and private enterprise in all its forms, and, in particular people, both as citizens and as consumers. The final goal of this change in V Programme in stimulating the involvement and the interplay between these economic actors (and in the employment of a broader range of instruments which will include, in particular, market-related incentives) is balancing the short-term benefit of individuals, companies and administrations and the longer-term benefits of society as a whole.

**Fig. 3:**
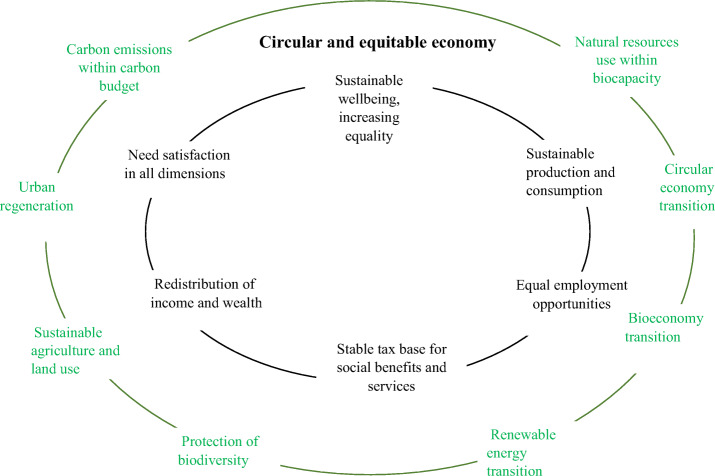
The circle of sustainable welfare state involving the policies for implementing the Green New Deal and the Circular economy within the commitments to the environmental and social goals in the sustainable development pattern. Adapted from Hirvilammi [11]

However, the adoption of more responsible patterns of production, consumption and living is not an easy task to initiate given the gap between the costs and the perceived (environmental) benefits in adopting such patterns [72]. The recognition of the gap is a key to put into practice the virtuous circle at the base of the “civil economy” and the related movements [73] as it is a potential cause of blocking or slowing the transition to CE [74]. On the other hand, being environment a public good this reflects a particular feature of sustainability transition [74] and in that framework also circular economy transition [14, 75, 76]. As a result, the adoption of Keynesians expansionary fiscal policies are necessary factors to switch the dynamo of the GDP growth embodying environmental and social goals and limits for building a new virtuous circle of a more sustainable welfare (Fig. 3). Contrary to the traditional circle of post-war period, the new virtuous circle should be redistributive guarantying the right to a decent income and the needed social services as well as more regenerative in the use of natural resources [11]. For this to happen it should incorporate the circular economy, bioeconomy and renewable energies transitions, the protection and enhancement of biodiversity to be perceived in the whole society (e.g. in urban centres) and sectors (in particular in agriculture) as a precondition for a more durable and sustainable development and a reduction of carbon emissions into identified limits to fight climate change.

## Discussion

In next sections, we discuss some of the key results evidencing the importance of the potential Keynesian contribution in the current market economy of many industrialised countries based on neoliberalism paradigm, with minimal state intervention and the need for moving towards the CE and sustainable development. This provides the base for discussing the new paradigm that we propose and its mechanisms in the framework of the existing literature dealing with the CE transition. Finally, we discuss the Global and Italian Green Deal as examples of topical political programmes to be stimulated by expansionary Keynesian fiscal policies as a precondition for starting a new virtuous circle of sustainable welfare needed to face the current multiple global challenges. The latter, forces global Governments to adopt “mission oriented” political plans [77] and in particular “green mission oriented” plans [78] and not whatever kind of expansionary fiscal policies to redistribute wealth and support the employment as in the Keynesian period.

### The Potential Keynesian Contribution to a Future Economic and Political Paradigm Underpinning the CE

In the previous sections, we briefly summarised some of the key issues of the contribution of John Maynard Keynes and its application in policies of many countries since the last century. We also outlined the effects of the reduction of the importance of Keynesian paradigm in policies and in the economy with the progressive dominance of the neoliberalism paradigm. This exercise has been made to highlight the implications of such a process of transition for the emergence of the global governance of the current multiple challenges (environmental, energy and food) making clearer other potential causes of the unsustainability of linear paradigm. The two paradigms have been extraordinarily influential and contrasting, nevertheless they were mainly functional to a linear economy approach.

The neoliberalism paradigm marked a deep change both to the Keynesian and to the classical paradigm [21]. In the classical liberalism, e.g. Adam Smith (the father of liberalism) foresaw an active role of the State in the economy and its intervention in the provision of a specific legislation (e.g. he recommended a reform of agrarian contracts), or essential public services for the society and its development such as primary public education as well as in avoidance monopolies in the market [24]. The rise of neoliberalism has been signed by a dismantling of welfare state in European countries [79, 80] even if not in an equal manner depending on the perceived causes of the oil economic crisis of the 1970s. For example, Britain underwent the dismantling of major Keynesian institutions and policies due to the fact that Keynesian paradigm was perceived as one of the main causes of the crisis whereas in France the neoliberal reform mainly focused on industrial policy due to the identification of the causes in the lack of competitiveness caused by the dirigiste policies [80].

The neoliberalism has become the dominant paradigm in the economy and in the political landscape of the recent decades even if the intrinsic limits of a market economy [40] are well known since the beginning of the last century thanks to Keynesian approach and other though schools that contributed to evidence such limits (e. g. neo-institutional theory, public choice school) [44, 48].

Keynes contributed to shed light to some of the limits of a market economy by finding solutions to the economic crisis and giving a new vigorous stimulus to the public intervention in social protection as an essential base for the modern welfare state in Europe [24] whereas Pigou [81] identified the presence of negative externalities arising from the economic activities and the possible remedies in the form of environmental taxes. After Pigou many scholars in the field of environmental economics as well as ecological economics further analysed the relationships between the natural environment and the economy [82]^9^. Contrary to environmental economics that have been mainly concentrated on market instruments to correct the environmental externalities [83], the ecological economists placed their critical analysis on the complex interplay between the natural ecosystems and the economy, on the concept of limits of the natural environment, on an intrinsic value of nature [72]. On a macroeconomics perspective Spash and Schandl [84] introduced the analysis of economic growth and environment, arguing that the approach by Post-Keynesian economics has similarities to the one of ecological economics but differs with the regard to the concept of “growth” that is not envisioned within the limits of natural environment. On the contrary, they underline that Keynes considered “capital accumulation as a means to an end not an end in itself”, evidencing that it was a temporary measure. “Avarice and usury and precaution must be our gods for a little longer still. For only they can lead us out of the tunnel of economic necessity into daylight” [85].

However, despite all these contributions the global economy continue to be confronted with old (environmental problems) and new negative effects (or externalities) of the market economy (exacerbated by the neoliberalism paradigm) such as the unemployment due to the delocalization, the high number of deaths on the workplace due to poor safety measures, the low wages leading to the so-called “working poors”, the negative effects derived from the increasing deregulation of financial markets, the decline of ethical values in the economy; the “crowding out” effect of market goods towards non-market goods having a scarce provision even if are key for the wellbeing of the contemporary society [40, 86]. Non-market goods such as air and water quality do not have a market or a price but clearly affect the opportunity of having a more healthy and good life of people. In that, the pollution induced effects on human health and damage to the Earth ecology are threating the sustainability of current and future generations [87]. Nevertheless, recent contributes have questioned the validity and the emergence of the link between growth and resources consumption highlighting that technological progress and productivity increases are allowing to produce more goods with less resources [88].

In the current market economy increasingly based on neoliberalism paradigm, with minimal state intervention and the need for moving towards the CE and sustainability, the development of voluntary initiatives of environmental and social innovations from companies or consumers is insufficient. This is due to the incapacity of the market of their recognition being featured by “market failures” [89] (in this case they are positive externalities) with the result that their provision is lower compared to the utility they have for the society. Moreover, bottom-up voluntary initiatives coming from the companies and consumers towards more sustainable and circular production and consumption models (and in general towards the so-called civil economy that we emphasised) are also challenged by market barriers generated by the competition with the conventional and less sustainable production and consumption models [14]. On consumers side, higher prices of green products and lower availability of green products in the market discourages environmentally conscious consumers favouring the conventional products [90] that have a lower price due to the discharge of environmental externalities to the society [89]. In the same line, voluntary initiatives of more cleaner and circular production models from the companies are challenged by the investment’s costs and the capital shortage [91, 92] as well as by a mainstream culture that does not see the eco-innovation as “win win” solution for the redesign of a company [93–95]. The study by Kirchherr et al., [93] shows that one of the most relevant barriers to the CE implementation is a “hesitant company culture” that relegate the knowledge of the concept of CE to environmental departments disregarding operative or financial departments whereas Rizos et al., [95] evidenced that in the business world, the CE concept is still largely unknown or mainly confined to waste management or recycling. This, of course depends on the orientation towards sustainability innovation among companies that could have different facets [96]. For example, it is in this perspective that we must frame the increasing phenomenon of greenwashing [97].

Clearly, these barriers could prevent the circular economy to shift towards a wider development [76] as happening by the renewable energy transition where in particular the role of incumbents seem relevant [73]. In the last decade renewable energies had a great development thanks to incentives policies carried out in European Union including Italy [98] as well as in other countries such as China [99, 100]. The Director of IRENA, commenting on the unprecedented successful acceleration of renewable energy development, evidenced the need for stronger enabling policies and a significant increase in investments over the next 10 years. Moreover, he evidenced the role of IRENA in promoting the knowledge exchange and the importance of collaboration with all the stakeholders both in the private and public sector leaders to accelerate the path of the energy transition towards a higher share of renewable energies [101]. In this view, also Sovacool [102] pointed out that energy transition is a collaborative effort in the whole society as “neither private markets nor government agencies seem likely to spur a transition on their own”. As a result, new instruments should be provided to consumers (other than price signals) and policy makers to innovate their choices [103].

These issues create a connection with the new economic and political paradigm underpinning CE transition that we proposed in Section Revisiting the Keynesian Paradigm in the Light of the Current Circular Economy Transition. The new paradigm entails a revisited Keynesian liberalism [21] and a more active role of economic agents: consumers [59] and companies by means of bottom-up instruments of environmental policies such the law of civil responsibility, moral persuasion and voluntary agreements between public administrations and industry [72]. The validity of this civil responsibility law is not intended to be restricted to the solution of environmental problems given that the latter are linked to social issues (Piketty, 2019). Indeed, the use of such instruments should be framed within a wider framework of top-down and bottom-up initiatives towards sustainable development which achievement entails the use of participative tools and a democratic dialogue within the actors in the society for the construction of new norms of social, educational, fiscal and climate justice [70, 104]. These norms will have to contrast with the present hyper concentration of economic power. On the contrary, the economy of the 21st century must be based on the permanent circulation of power, wealth and knowledge [104].

Finally, the creation of a “virtuous circle of sustainable welfare state” has been proposed by Hirvilammi [11] as a reframe in view of the current multiple challenges of the traditional “virtuous circle of welfare state” implemented in the post-war period in particular in Europe.^10^ The concept of “virtuous cycle of welfare state” centred on the Keynesian paradigm (“economic-growth oriented”) has been also supported by the economist-sociologist Gunnar Myrdal and his theory of circular cumulative causation (CCC) where he emphasises the concept of disequilibrium as a feature of market economy as well as of the existence of a strict interrelation between the elements of social and economic processes. In that, Myrdal emphasised the role of the state in regulating the market economy towards the full employment as theorised by Keynes. Hirvilammi [11] highlights that the notion of virtuous circle in Myrdal was closely associated to values and ethical norms leading to a “virtuous circle between economy, politics and ethics”. This approach acquired the ideological status in the political discourse in particular in Nordic countries such as in Finland.

The “virtuous circle of sustainable welfare” looks beyond economic growth compared to the traditional to embrace a welfare state that relies on a regenerative and distributive economy able to ensure wellbeing as well as take into account the need for limiting the environmental impacts of economic activities. Such “virtuous circle” is centred on the concepts of “sufficiency” for production and consumption activities, “circular economy” as a means to reduce the demand for materials and amount of waste, “planetary boundaries” as a constraint to the functioning of human activities, and “regenerative and distributive” economy for providing good jobs and decent standard of living.

Our study can be considered an integration of the creation of this “virtuous circle of sustainable welfare” as it tries to shed light on the mechanisms that could be useful to achieve such political and societal goal and move beyond the neoliberalism paradigm. Moreover, such a virtuous circle, fuelled by a law of civil responsibility among all the stakeholders (according top-down and bottom-up approach) is representative of the policy actions and the decision-making processes that several countries are adopting to overcome the Covid-19 emergence.

### Expansionary Keynesian Fiscal Policies in the Circular Economy According to the Global Green New Deal

Jackson [31] evidences that after the economic crisis of 2008, the discussion about the adoption of expansionary policies in the spirit of Keynes involved both the public stimulus to the traditional and “old extractive economy” as well as to the green economy and the sectors of “Green New Deal”.

The idea of “Green New Deal” has been proposed in 2007 during a meeting in London of some environmentalists and economists [8] and later in a report commissioned by the UNEP [105] in response to the great recession of 2008 (that followed the previous crisis of 2000, so called dot-com-bubble, caused by the high speculation in web companies at the end of 1990s) [106], where the world was confronted by another big financial and economic crisis combined with other crisis concerning food security and fossil-fuels dependence. These multiple crises, according to the report, should have required the same kind of interventions of the New Deal developed by the US President Roosevelt in the 1930s and adapted on the global perspective of a sustainable development. The main goals of the “Global Green New Deal” were the economic recovery and the creation of jobs, the reduction of environmental problems (carbon dependency, ecosystem degradation, water scarcity) and the achievement of Millennium Development Goals of ending extreme world poverty by 2015 [105]. The Report also pointed out that US$ 3.1 trillion in economic stimulus packages should have been mainly pushed by G20 countries in favour of green investments in the following five main areas:

- Energy efficiency in old and new buildings;
- Renewable energy technologies, such as wind, solar, geothermal and biomass technologies;
- Sustainable transport technologies, such as hybrid vehicles, high speed rail and bus rapid transit systems;
- The planet’s ecological infrastructure, including freshwaters, forests, soils and coral reefs; and
- Sustainable agriculture, including organic production.

Within these areas the [105] recommended G20 countries to prioritise the investments on improving energy efficiency in new and existing buildings, stimulating renewable energy sources and enhancing sustainable transport.

In the year of the crisis, the idea of massive public investments according to Keynes (as evidenced previously) was abandoned in favour of the adoption of austerity fiscal policy programmes keeping latent the idea of a Green New Deal [8, 31]. G20 and others (e.g. in Latin America, see the case of Chile, [106, 107] provided public stimulus for the sectors in the renewable energies transition [108] and green economy as in EU [109]. However, Asian countries such as China and South Korea have invested (with more ambitious plans) about 3–5% of their GDP to the development of industries related to the technologies of solar panels, electric cars and wind turbines. Even if there was not a shared and systematic plan at the global level with policies involving massive investments, in those countries where the latter have been applied, they produced important results such as in China [99]. As opposite, US have devoted less of its GDP (0.9%) leaving the Green New Deal to develop with lower public spending [109].

On 2018, at the global level, the whole renewable energy sector employed 11 Million of people, with an increase by 6.79% compared to the year 2017. The solar photovoltaics (PV) is the compartment with the highest amount of workforce accounting of one third of the total. Most of the workforce is concentrated in some countries such as China, Brazil, USA, India and some member States of EU [110]. Moreover, over time in terms of renewable energy technology, the solar PV recorded constant improvement in the material and energy efficiency of PV cells and panels as well as better life-cycle energy and environmental performances [111].

The prices of electricity from renewables and in particular from solar PV, have strongly reduced since 2010 being competitive (without financial support) with the price of conventional power sources [112]. The Levelized Cost of Energy (LCOE) of utility-scale solar PV fell by 73% between 2010 and 2017, to USD 0.10/kWh due to the decrease by 81% of solar PV module prices since the end of 2009 as well as the reductions in balance of system (BoS) costs [112].

Within the green economy transition, the European Union (2019b) has supported several projects over the years that can be framed with the so called current “European Green Deal” [113]. Table 1 lists some of these projects funded by UE. We can see that, within the cohesion policy funding the EU provided new economic opportunities in former mining towns, by means of European Investment bank and European Fund for Strategic Investments provided funds for helping citizens and businesses to reduce CO_2_ emissions and energy bills and so on [109]. Moreover, recently (January 2020) the European Commission presented to the EU Parliament the legislative proposal of the plan intended to invest 1 thousand billions of euros in the next 10 years. The goal is transforming the EU in the first green continent at the global level [114]. The proposal aligns with the EU Parliament approved resolution of November 2019 that declared a climate and environmental emergency in Europe and globally as well as that all relevant legislative and budgetary proposals of EU Commission should meet the objective of limiting global warming under 1.5 °C [115].

**Table 1:** EU funded projects in the sectors of the European Green Deal. Source of the data: European Commission, January 2020

Goal of project	Activities	Country/s	Policy funding
Creation of new economic opportunities in former mining towns	• Transformation of a former coal mine into a cultural area including a museum, a congress centre and a new concert hall; • Creation of opportunities in construction, tourism, cultural and food services sector.	Poland	Cohesion policy funding
Helping citizens and businesses to cut CO_2_ emissions and reduce energy bills	• Installation of solar panels on private homes; • Renovation of multi-apartment buildings; • Energy efficiency investments in industrial companies.	Lithuania	European Investment Bank guaranteed by the European Fund for Strategic Investments
Investments in new environmentally friendly technologies	• Substitution of harmful refrigerants in commercial refrigerators to reduce GHGs, increase the energy efficiency and reduce the costs.	Italy, Spain and Romania	EU’s LIFE programme
Reskilling of workers from coal industry regions	• Provision of training in welding; • Teaching to handle of machines such as fork-lift trucks; • Help workers to obtain a driving licence for small trucks and lorries.	Czechia (Czech Public Employment Service in Nord-Moravia)	European Social Fund
Reduction of car emissions	• Reduction of the weight of vehicles on the road by replacing heavier car manufacturing materials with lighter and renewable components.	Poland and Italy	EU’s LIFE programme
Support to social housing	• Building 524 affordable and energy efficient social housing units	Spain	European Investment Bank guaranteed by the European Fund for Strategic Investments

### The Green Deal in Italy: a Case Study

**Fig. 4:**
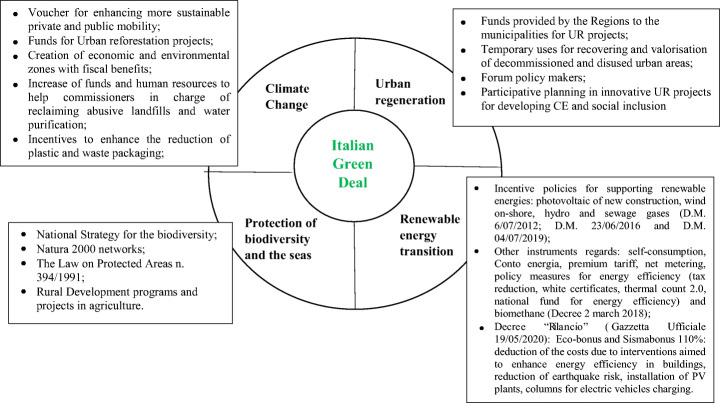
The main building blocks of the Italian Green Deal. Sources of data: Climate change: [118]. Urban regeneration: [119–121]. Renewable energy transition: [122–127]. Protection of biodiversity and the seas: [128–130]

The concepts behind the Green Deal are recurrent in particular since the 2019 in Italy, where according to the new Government the adoption of an Italian Green Deal will reorient the whole Italian productive system towards a more sustainable development. In that vision Italy would play a leading role at the global level [116]. The Green Deal promotes the development of renewable energies, the urban regeneration, the protection of biodiversity and the seas, and addresses climate change [117]. Figure 4 outlines the four main measures and tools for the different sectors of the Italian Green Deal which are discussed in the following.

### Urban Regeneration

In Italy, urban regeneration (UR) is a goal mainly perceived by the Regional Governments. Italy does not have yet a national urban policy [131] even if a recent Law (n. 56 of 7 April 2014) modified the provincial administrative system to give to some large provincial cities greater responsibilities. In this view, the Law, on “Metropolitan Cities, Provinces, Municipal Unions and Municipal Mergers” created 10 Metropolitan Cities: Torino, Milano, Venezia, Genova, Bologna, Firenze, Bari, Napoli, Reggio Calabria, and Roma Capitale. These cities replaced their corresponding provinces and provincial governments with a metropolitan government led by a President and represented by the Mayor of the metropolitan area’s primary city [131].

Compared to the past, UR, is receiving an increasing attention in the current regional policies also in response to the citizens demand of public policies for a better quality of life**.** Moreover, its role is considered strategic in the framework of local policies for the improvement of the competitiveness of cities and territories and achievement of a more sustainable development. By perceiving UR, local policies promote the attractiveness and regeneration of existing building stock according to the principles of sustainability [119]. However, the concept of UR is not only linked to the conservation and recovery of the existing building stock as UR entails a sustainable and circular innovation process into the social, cultural and environmental heritage of a city [132, 133]. In this view, UR in a CE perspective conceives cities as “living organism” with its own dynamics within the common evolution pattern of born, grow, stabilisation, decline and die [132].

#### Environmental and Socio-economic Impacts of UR Projects.

The implementation of UR projects is a driver for socio-economic development not only for the local scale (e.g. the town) where the project is implemented but also for the surrounding territories, the whole region [134] and in case of very large projects (such as e.g. the Expo of Milan 2015) the whole national economy [135].

Successful projects of UR implemented in Italy at local level include the project of Bari in Southern Italy which provided financial support for opening new individual enterprises in craftwork and tourist sectors as well as the offer of new accommodations for university students. Moreover, the project contributed to crime prevention and public safety by the upgrading of street lighting and the adoption of three new “24 hour” centres run by volunteers. In that perspective, UR, by acting on the valorisation of the cultural or man-made/natural heritage of cities, promotes the use of endogenous activities favouring their reintroduction into the socio-economic process according to the principles of the CE [136]. The project of the city of Pozzuoli (Naples) for the future socio-economic revaluation of an abandoned industrial area is part of the same conceptual and circular framework as by a participative process involving the stakeholders of the city, the development of cultural activities (urban equipped areas, sport centres, parks areas and accommodation centres) results the most suitable options and the main driver for the revaluation of an old industrial area [137] or in general for historical cities [133]. The findings of this latter study show the opportunity of combining CE transition and historical urban landscape conservation by means of the adoption of cultural projects involving three main typologies of stakeholders: Universities, cultural heritage entrepreneurship and local authorities. In that, the creation of these synergies contributed in 2005 to achieve for the city of Syracuse, along with the Ortigia island the status of World Heritage Site by UNESCO [133].

### Renewable Energy Transition in Italy

Renewable energies sources (RES) in Italy, thanks to different factors such as incentive policies [98] and technological innovation, have become in the last years one of the most important sources for the generation of primary energy in Italy [138]. On 2018, the RES had a share by 34% in the whole electricity produced whereas for heat they had a share by 19.8% in the whole mix of sources for heat generation [139]. In transport sector the RES share is lower being 6.5% of the whole sources of the mix [139]. In 2017, the production of RES contributed to satisfy a share by 18.3% in gross final energy consumption. As a result, Italy surpassed by 17% the target for the country share of energy from renewable sources that have been established in accordance to the directive 2009/28/CE (Directive 2009/28/EC). In terms of employment, not very recent data related to the period between 2008 and 2011 show that e.g. in the solar PV sector the number of employees in Italy shifted from 2300 to 18000^11^.

In Italy the average production price of electricity from PV (considering a production of 1300 kWh/kwp/year in central Italy) ranges between 0.04 Euro/kWh and 0.08 Euro/kWh (with/without a tax deduction of 50%) for the PV plant^12^.

Within the policy measures for supporting electricity from renewable sources three ministerial decrees such as D.M. 6/07/2012; D.M. 23/06/2016 and D.M. 04/07/2019 have been adopted over time in Italy for regulating the incentive mechanisms to accelerate the development of renewable energies sources (RES) for small, medium and large plants. The type of plants that are eligible of the incentive policies are the following: photovoltaic of new construction, wind on-shore, hydro and sewage gases [140].

The incentives are provided to private subjects, companies and public administrations and depending on the size of technology the mechanisms consist in the form of feed-in tariffs and alternative mechanisms based on the plant capacity [141].

Other instruments are also available in support to the development of renewable energy sources for the production of electricity [140] and evidenced in Fig. 4.

#### Environmental and Socio-economic Impacts of Renewable Energy Transition.

As evidenced above, the supporting policies to RES contributed to accelerate the renewable energy transition in Italy [98] helping the country to reach the Europe’s decarburisation targets in line with the Paris Agreement as well as improving Italy’s competitiveness due to the diversification of the energy sources in the Italian energy mix. The benefits relate to the reduction of the gap between European and Italian energy prices and the improvement of security and flexibility of energy supply [141]. The diversification also reduced the negative effects on the economy that could derive from the volatility of the price of fossil fuels as well as the negative environmental and health externalities coming from non-renewable energy generation [142]. In that, the development of renewable energies agrees with the definition of sustainable development in both environmental and socio-economic terms by avoiding to shift to the future generation’s further burdens [142].

### The Protection of Biodiversity and the Seas

Biodiversity is considered an essential and imperative factor for the global economies facing the multiple challenges of the sustainable development. In that, biodiversity contributes to make more sustainable and resilient economic activities such as agriculture and forestry as well as the urban landscape of cities [128].

Italy has one of the most relevant heritage of species in Europe both in terms of total number and high rate of endemism. To the building of such heritage several factors contributed including its cultural history and central location in the Mediterranean Basin (one of the 33 biodiversity hotspots in the world). In Italy, in an area that is 1/30 of the European continent, are concentrated more than 30% of animal species and almost 50% of plant species available in Europe. As many species are at risk of extinction, due to anthropic activities, their protection is an effort that should be addressed at societal level to reconcile their deployment within the principles of sustainability [129].

#### Environmental and Socio-economic Impacts of Improving Biodiversity.

The National Strategy for the Biodiversity has undergone during the years a monitoring activity aimed to evaluate its implementation. The third assessment report of year 2019 evidences the progresses of Italy towards each National target and the many initiatives adopted in each working area. In the achievement of the priorities in 15 working areas many activities in progress (59%) whereas others are started (24%), implemented (7.5%), not evaluated (9%) and not yet evaluated (0.5%) [130].

Agriculture is one of the most involved sectors in terms of projects and initiatives related to biodiversity [143], as shown by the “Natura 2000 and Rural Development programs” and the attention given to the food-biodiversity link during EXPO 2015 [130]. In this view, in Italy since more than 20 years ago, e.g., the wine sector invested in the research and reuse of old varieties. This has provided a relevant competitive advantage to the country which economic value of the exports of wine and its quality have continuously increased over the last decade whereas the quantity exported has been stable [144].

### Climate Change

The last year the Governments has adopted the so called “Climate decree”^13^ as a first step to contrast more strongly the climate change and also improve air quality. Unfortunately, Italy, both at the global level and in Europe records every year a very high number of premature deaths from exposure to PM2.5 fine particulates.

The last report about the analysis of climate indicators in Italy published by the ISPRA [145], evidences that on 2018 in Italy the annual average temperature achieved a new record with a deviation by +1.71 °C compared to the average value of the period 1961–1990. On 2018 all the months (except February and March) have recorded temperatures higher than the average temperature of the period. The minimum daily average temperature achieved a new high record (+1.68 °C) compared to the record in average temperatures of the year 2014 (+1.58 °C).

The average rainfall, on 2018, showed values moderately higher than the average. However, the pattern of rainfall has been discontinuous and very wet months have been followed by dried months. The highest daily values of cumulated rainfall have been recorded in the year 2018 in three Italian regions (Liguria, Friuli Venezia Giulia and Calabria) in October where in some days (4th, 27th, 28th) the cumulated rainfall ranged between 300 and 400 mm [145].

The Climate decree devotes financial resources to a wide range of measures (some of them are summarised in Fig. 4) in the forms of incentive, voucher, funds intended for example to sustainable public and private mobility, reforestation, green corners, reduction of plastic and waste, improvement of air quality.

## Conclusions

This study briefly analysed the evolution of the Keynesian paradigm for the purpose of highlighting its main features and how it could be useful in view of the CE transition and the need for facing the global multiple challenges related to the environment, energy and food. The Keynesian paradigm mainly adopted in industrialised countries (such as in Europe) after the Second World War lead to the creation of the welfare state and a period of high economic growth, employments rates and increased well-being in a context of growing use of natural resources. Moreover, in such period at international level important agreements have been promoted by the United Nations with the purpose of achieving social, humanitarian, health and environmental goals. The Keynesian paradigm has been progressively replaced since 1980s by the neoliberalism paradigm that redesigned the State intervention in the economies of industrialised countries to a idifferent extent as well as the governance of social, humanitarian, health and environment issues at the global level. The central feature of the neoliberalism is the strong focus on the market economy as a mean to satisfy the wellbeing of the society without considering the resource constraints. However, the wellbeing of the society depends on many goods and services that do not have a market price such as the environmental quality, a decent job, healths, housing and educational systems and so on. This calls for the urgent adoption of a new paradigm underlying the current cultural, economic, environmental, political and social landscapes. We emphasised a model of economy underpinning CE transition based on a revised Keynesian paradigm that implies a redesign of the current role of the State intervention as well as the role of responsible consumers, companies and institutions with the purpose of creating a virtuous cycle between these three fundamental actors. Given the dominant neoliberalism paradigm and its limits, such “virtuous cycle” can be triggered and implemented only by a political intervention as civil responsibility actions and practices are costly. This explains why the number of responsible consumers and companies is still low and is not able to widen their share on the basis of their market power. However, the reliance of an active role and a higher responsibility of the consumers and companies is essential to assure that the political intervention occurs according to the social, environmental and economic goals. In our view, in such a framework the Green New Deal that we evaluated as a kind of Keynesian expansionary policy has the potential to break the wall of the neoliberalism paradigm and create the bases for the “virtuous circle of the sustainable welfare” which also involve and trigger the transition to a circular economy and in general a more sustainable wellbeing within the limits of the natural environment. Nowadays, Keynes famous sentence “The government should pay people to dig holes in the ground and then fill them up” should be revisited “The Government should pay people to dig holes and plant trees instead of filling simply them up again”.

## Footnotes

1 “(T)he ideas of economists and political philosophers, both when they are right and when they are wrong, are more powerful than is commonly understood. Indeed, the world is ruled by little else. Practical men, who believe themselves to be quite exempt from any intellectual influences, are usually the slave of some defunct economist.”John Maynard Keynes, *The General Theory of Employment, Interest and Money* p. 383.

2 For semplicity sake this study groups the terms "neoliberism" or "new liberalism" under the term of “neoliberalism.”

3 Striving to be the first climate-neutral continent. https://ec.europa.eu/info/strategy/priorities-2019-2024/european-green-deal_en

4 Web of Science: https://apps.webofknowledge.com/UA_GeneralSearch_input.do?product=UA&SID=F5MuwfvFZ3dVF3ImzQe&search_mode=GeneralSearch

5 Keynes suggested the need to reject Say’s law of markets and for positive intervention by government to encourage aggregate demand to restore equilibrium, with unused under employed supply possibilities. At this time the wide spread unemployed workers, machines, and immense surplus stocks of coal above ground and other raw materials, were apparent for all to see. He suggested that the government rather than run surpluses or balance budgets prepare to run a deficit .... a proposal against all canons of sound finance of neoclassic doctrines. It is important to recognise that during the interwar period when Keynes was most prolific, the economy was afflicted by persistent under-use of virtually all real resources. It was therefore reasonable to assume the supply of labor, capital, and raw materials were in great abundance given the high degree of under employment that obviously existed. Thus, a small increase in price would bring onto the market any amount of real resources of capital, raw materials, fuel and labor necessary.

6 The new liberalism shared with the classical liberalism the features of individual autonomy, private property, and limited government intervention (Furner, 2001).

7 http://treccani.it/enciclopedia/l-economia-civile_(Il-Contributo-italiano-alla-storia-del-Pensiero:-Economia)/

8 https://sfenvironment.org/consumer-responsibility

9 For a comprehensive review of the value of nature and the contribution of the different scholars from pre-classical, classical, neoclassical and environmental economics until the years 2000 see Gómez-Baggethun et al., 2010.

10 Even if the concept date backs to the 1930s where there were also the initial political and social responses (such as the creation of International Labour Organization, ILO) to the liberal tradition and “laissez-faire” ideology.

11 https://www.fotovoltaiconorditalia.it/mondo-fotovoltaico/sviluppo-fotovoltaico-benefici-occupazione

12 https://www.fotovoltaiconorditalia.it/idee/quanto-costa-elettricita-prodotta-da-fotovoltaico

13 https://www.minambiente.it/comunicati/decreto-clima-un-primo-importante-passo-contrastare-i-cambiamenti-climatici
